# Correction: Home range and activity budget in the Falkland Steamer Duck (*Tachyeres brachypterus*)

**DOI:** 10.1371/journal.pone.0345472

**Published:** 2026-03-19

**Authors:** Alix M. I. Kristiansen, Alastair M. M. Baylis, Sébastien A. P. Dupray, Luc Lens, Heather Q. Mathews, Lucy J. Mitchell, John P. Y. Arnould

In the Study site and animal handling procedures subsection of the Methods, there is an error in the fifth sentence of the first paragraph. The correct sentence is: The density of Falkland Steamer Ducks was estimated to be of 4.3 pairs·km^-1^ along the coastline of Stanley Harbour and its surroundings (Fig 1, [35]).

In the Home and core ranges subsection of the Results, there is an error in the seventh sentence of the first paragraph. The correct sentence is: For example, northern Bleaker Island supported a higher breeding pair density (9.8 pairs.km^-1^) compared to Stanley (4.3 pairs.km^-1^), which could reflect individuals occupying smaller, resource-rich territories (Kristiansen et al in prep.).
