# Supplementary material for: Home range and activity budget in the Falkland Steamer Duck (Tachyeres brachypterus)
Source: PLoS One. 2025 Oct 7;20(10):e0333302. doi: 10.1371/journal.pone.0333302 (PMC12503321; doi:10.1371/journal.pone.0333302)
Supplement: S1 Fig — Following Calabrese et al (2016) recommendation, because the curve does not reach an asymptote, the showed individuals were excluded. (DOCX) [file pone.0333302.s001.docx]

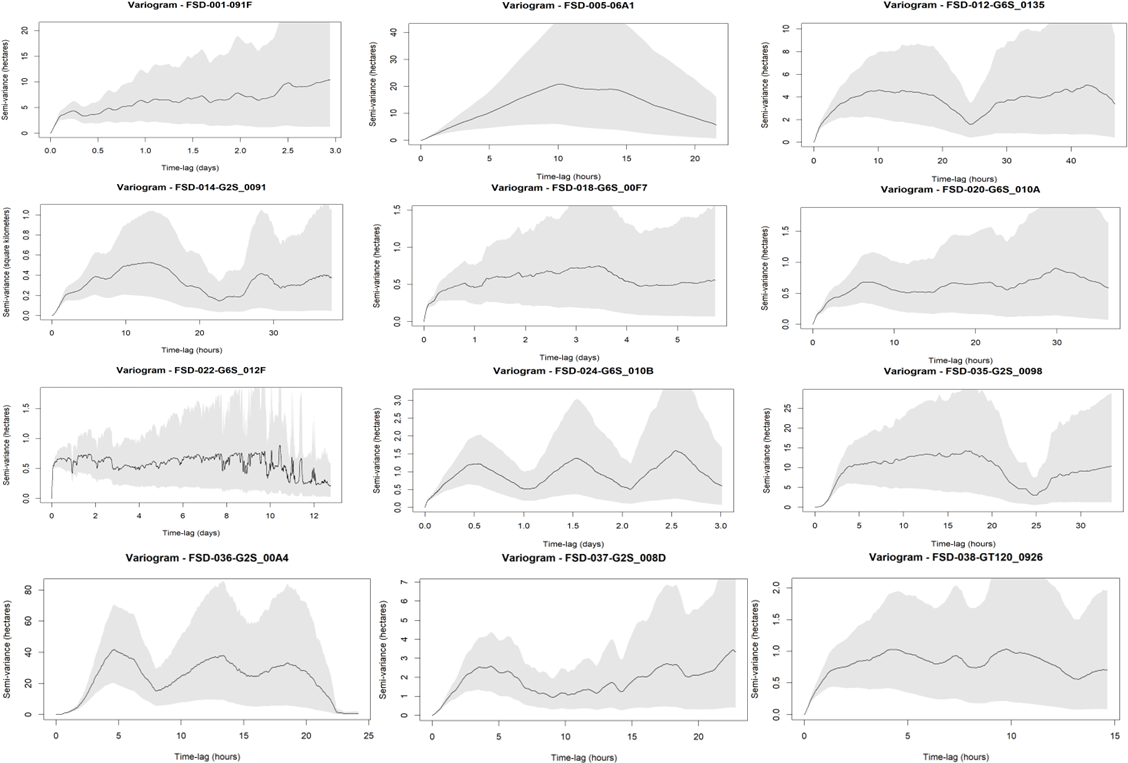


**S 1 Variograms for individuals with insufficient data to estimate home ranges.** Following Calabrese et al (2016) recommendation, because the curve does not reach an asymptote, the showed individuals were excluded.
