# Supplementary material for: Home range and activity budget in the Falkland Steamer Duck (Tachyeres brachypterus)
Source: PLoS One. 2025 Oct 7;20(10):e0333302. doi: 10.1371/journal.pone.0333302 (PMC12503321; doi:10.1371/journal.pone.0333302)
Supplement: S1 Table — (1/id) notation represents individual as a random effect. Bold models are single models with the best score, or multiple models used for model averaging (those within delta AIC = 4). Null models (i.e., no covariates) presented in italics. (DOCX) [file pone.0333302.s003.docx]

**S 3 Table AIC and delta AIC for models explaining (i) travelled distance, (ii) home range, (iii) core range, (iv) proportion of time spent on land, (v) proportion time foraging**. (1/id) notation represents individual as a random effect. **Bold** models are single models with the best score, or multiple models used for model averaging (those within delta AIC = 4). Null models (i.e. no covariates) presented in italics.

| Dependent variable | Model type and structure | AIC | ΔAIC |
| --- | --- | --- | --- |
| (i) Home range (95 %) – sex | **Size ~ area kelp UD_95_ + study site** | **46.7** | **0.00** |
|  | **Size ~ area kelp UD_95_** | **50.5** | **3.79** |
|  | **Size ~ area kelp UD_95_ + study site + sex** | **50.6** | **3.92** |
|  | **Size ~ area kelp UD_95_ + study site + percentage kelp UD_95_** | **50.7** | **3.93** |
|  | *Size ~ null* | 61.3 | 14.58 |
| (i) Home range (95 %) – status | **Area kelp UD_95_ + study site** | **36.9** | **0.00** |
|  | *Size ~ null* | 47.2 | 10.32 |
| (ii) Core range (50 %) – sex | **Size ~ area kelp UD_50_ + study site** | **41.5** | **0.00** |
|  | **Size ~ area kelp UD_50_ + study site + percent kelp UD_50_** | **42.1** | **0.61** |
|  | **Size ~ area kelp UD_50_ + study site + sex** | **45.2** | **3.68** |
|  | *Size ~ null* | **57.5** | **15.98** |
| (ii) Core range (50 %) – status | **Size ~ area kelp UD_50_ + study site** | **32.3** | **0.00** |
|  | **Size ~ area kelp UD_50_** | **32.3** | **0.01** |
|  | **Size ~ area kelp UD_50_ + study site + percent kelp UD_50_** | **33.8** | **1.56** |
|  | **Size ~ area kelp UD_50_ + percent kelp UD_50_** | **34.7** | **2.41** |
|  | **Size ~ area kelp UD_50_ + status** | **35.1** | **2.86** |
|  | *Size ~ null* | 51.5 | 19.25 |
| (iii) Distance per day | **Distance~ dist to kelp + dist to structure + study site + status + (1/id)** | **1119.8** | **0.00** |
|  | **Distance ~ dist to kelp + dist to structure + (1/id)** | **1120.5** | **0.63** |
|  | **Distance~ dist to kelp+ dist to structure + (1/id)** | **1122.0** | **2.12** |
|  | *Distance ~ null* | 1271.2 | 151.36 |
| (iv) Proportion time on land | **Prop.land ~ status + (1/id)** | **5367.2** | **0.00** |
|  | **Prop.land ~ status** + **(1/id) + study site** | **5367.3** | **0.15** |
|  | **Prop.land ~ null** | **5365.5** | **3.44** |
| (v) Proportion time foraging | **Prop.forage ~ areaKelp50 + areaKelp95 + dRoads + dSettlement + study site + percKelpUD95 + status** | **5942.02** | **0.00** |
|  | **Prop.forage ~ areaKelp50 + areaKelp95 + dRoads + dSettlement + study site + percKelpUD95 + status+ percKelpUD50** | **5943.08** | **1.05** |
|  | **Prop.forage ~ areaKelp50 + areaKelp95 + dRoads + dSettlement + study site + percKelpUD95 + status + dKelp** | **5943.99** | **1.97** |
|  | **Prop.forage ~ areaKelp50 + areaKelp95 + dRoads + dSettlement + study site + percKelpUD95** | **5944.07** | **2.04** |
|  | **Prop.forage ~ areaKelp50 + areaKelp95 + dRoads + dSettlement + study site + percKelpUD95 + status + sex** | **5944.09** | **2.07** |
|  | **Prop.forage ~ areaKelp50 + areaKelp95 + dRoads + dSettlement + study site + percKelpUD95 + status + percKelpUD50 + dKelp** | **5944.82** | **2.80** |
|  | **Prop.forage ~ areaKelp50 + areaKelp95 + dRoads + dSettlement + study site + percKelpUD50** | **5944.95** | **2.93** |
|  | **Prop.forage ~ areaKelp50 + areaKelp95 + dRoads + dSettlement + study site + percKelpUD95 + statuspercKelpUD50 + sex** | **5945.00** | **2.97** |
|  | **Prop.forage ~ areaKelp50 + areaKelp95 + dRoads + dSettlement + study site + status + percKelUD50** | **5945.29** | **3.27** |
|  | **Prop.forage ~ areaKelp50 + areaKelp95 + dSettlement + percKelpUD95 + status** | **5945.38** | **3.35** |
|  | **Prop.forage ~ areaKelp50 + areaKelp95 + dRoads + dSettlement + study site + status** | **5945.58** | **3.56** |
|  | **Prop.forage ~ areaKelp50 + areaKelp95 + dRoads + dSettlement + study site + percKelpUD95 + sex** | **5945.64** | **3.62** |
|  | **Prop.forage ~ areaKelp50 + areaKelp95 + dRoads + dSettlement + study site + percKelpUD50 + sex** | **5945.832** | **3.81** |
|  | **Prop.forage ~ areaKelp50 + areaKelp95 + dRoads + dSettlement + study site + percKelpUD95 + percKelpUD50** | **5946.02** | **4.00** |
|  | *For.prop ~ null* | 5971.772 | 29.75 |
