# Supplementary material for: Home range and activity budget in the Falkland Steamer Duck (Tachyeres brachypterus)
Source: PLoS One. 2025 Oct 7;20(10):e0333302. doi: 10.1371/journal.pone.0333302 (PMC12503321; doi:10.1371/journal.pone.0333302)
Supplement: S3 Fig — Colours represent the different behaviours (grey: travelling; green: foraging; blue: resting) for each breeding status (CR: chick-rearing; IF: incubating female; NB: non-breeding; PM: patrolling male). (DOCX) [file pone.0333302.s004.docx]

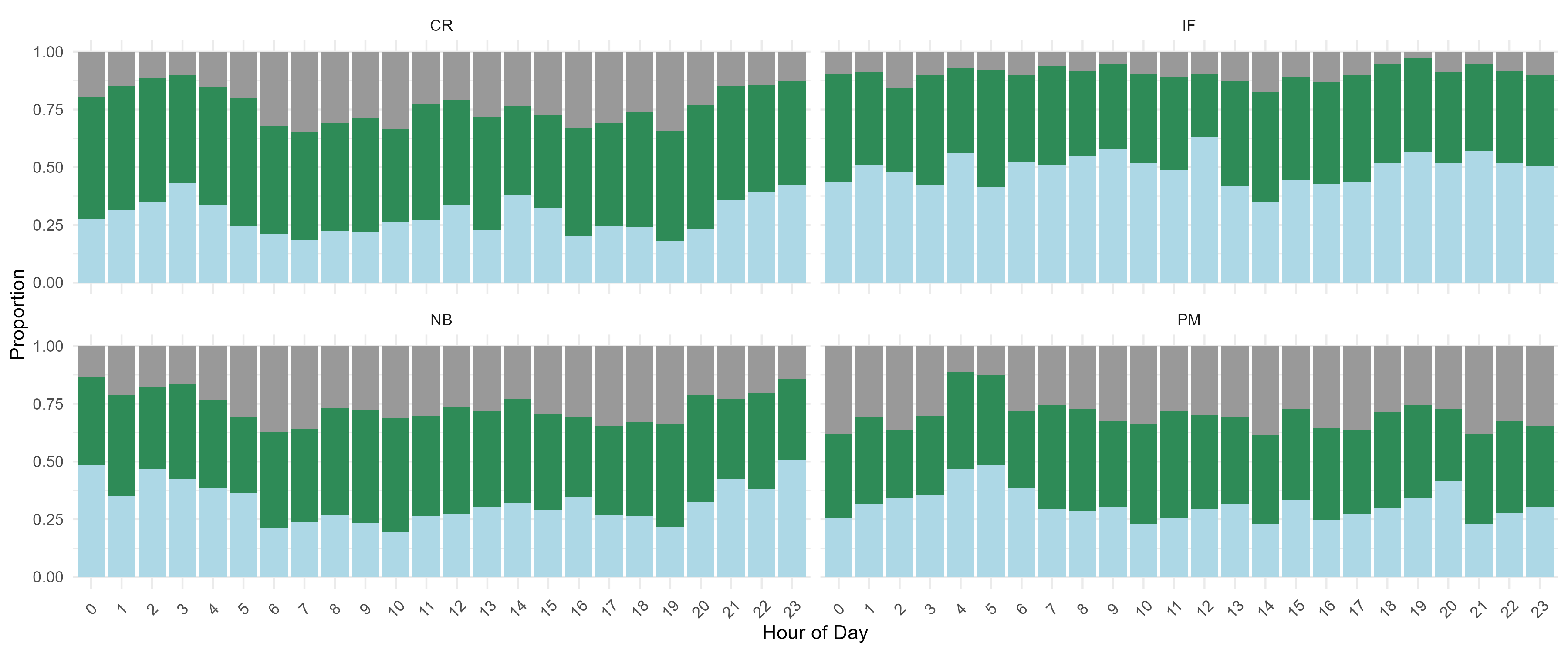


**S 4 Fig. Activity budget of the Falkland Steamer Duck.** Colours represent the different behaviours (grey: travelling; green: foraging; blue: resting) for each breeding status (CR: chick-rearing; IF: incubating female; NB: non-breeding; PM: patrolling male).
